# Ultrasound assisted phytochemical extraction of persimmon fruit peel: Integrating ANN modeling and genetic algorithm optimization

**DOI:** 10.1016/j.ultsonch.2024.106759

**Published:** 2024-01-06

**Authors:** Souvik Giri, Kshirod Kumar Dash, GVS Bhagya Raj, Béla Kovács, Shaikh Ayaz Mukarram

**Affiliations:** a Department of Food Processing Technology, Ghani Khan Choudhury Institute of Engineering and Technology, Malda, West Bengal, India; b Faculty of Agriculture, Food Science and Environmental Management Institute of Food Science, University of Debrecen, Debrecen 4032, Hungary

**Keywords:** Persimmon peel, Ultrasonication, Artificial neural network, Kinetics

## Abstract

In the present study, ultrasound assisted extraction (UAE) of phytochemicals from persimmon fruit peel (PFP) was modeled using an artificial neural network (ANN) and optimized by integrating with genetic algorithm (GA). The range of process parameters selected for conducting the experiments was ultrasonication power ($X_{U}$) 150–––350 W, extraction temperatures ($X_{T}$) 30–––70 °C, solid to solvent ratio $(X_{S}$) 1:15–––1:35 g/ml, and ethanol concentration ($X_{C}$) 40–––80 %. The range of responses total phenolic content ($Y_{P}$), antioxidant activity ($Y_{A}$), total beta carotenoid ($Y_{B}$) and total flavonoid content ($Y_{F}$) at various independent variables combinations were found to be 7.72–––24.62 mg GAE/g d.w., 51.44–––85.58 %DPPH inhibition, 24.78–––56.56 µg/g d.w. and 0.29–––1.97 mg QE/g d.w. respectively. The modelling utilised an ANN architecture with a configuration of 4–12-4. The training process employed the Levenberg–Marquardt method, whereas the activation function chosen for the layers was the log sigmoid. The optimum condition predicted by the hybrid ANN-GA model for the independent variables, $X_{U}$, $X_{T}$, $X_{S}$ and $X_{C}$ was found to be 230.18 W, 50.66 °C, 28.27 g/ml, and 62.75 % respectively. The extraction process was carried out for 25 min, with 5-minute intervals, at various temperatures between 30 and 60 °C, to investigate the kinetic and thermodynamic characteristics of the process, under the optimal conditions of $X_{U}$, $X_{S}$ and $X_{C}$. The UAE of phytochemicals from persimmon peel followed pseudo second order kinetic model and the extraction process was endothermic in nature.

## Highlights

• Persimmons' orange and red hues come from beta-carotene pigments.
• Persimmons peel phytocompound extraction was modeled using ANFIS.
• Effective diffusion coefficient at 30 °C extraction temperature was $3.187\times 10^{-11}$.
• Negative Gibbs free energy changes indicated thermodynamically spontaneous extraction.

## Graphical abstract

**Figure:**
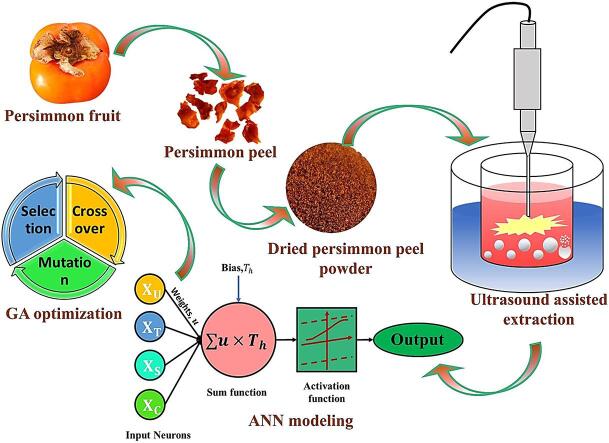

## 1 Introduction

Persimmons are the fruits grown on trees belonging to the Diospyros genus of the Ebenaceae family [1]. Astringent and non-astringent are the two types of persimmon fruit commercially available and the main attribute that makes the fruit attractive to consumers is its dark red peel color [2]. Persimmon is categorised as a climacteric fruit that contains bioactive substances such as phenolic compounds, carotenoids, and flavonoids, as well as primary metabolites, minerals, and vitamins [3], [4]. Approximately 10–12 % of the entire fruit gets thrown away as waste in the form of peel. This waste contains health-promoting substances such as phenolic compounds, carotenoids, and flavonoids, which possess antioxidant properties [1].

The peel of persimmon fruit contains a diverse range of substances, such as bioactive compounds, fibres, and antioxidants. The primary constituents commonly present in persimmon fruit peel comprise dietary fibre, carotenoids, flavonoids, phenolic compounds, vitamin c, tannins, and minerals such as potassium and manganese. Utilizing biomass wastes or by-products from the food, agricultural, and forestry sectors has recently attracted interest due to the recovery of health benefit compounds and the ability for these compounds to create value-added food products [5]. The substances derived from plant sources can be isolated from the waste by the extraction process, which involves the use of a chemical solvent and elevated temperature [6]. Various conventional techniques are employed to extract bioactive compounds from natural sources such as plants, fruits, and herbs. These procedures include solvent extraction, steam distillation, soxhlet extraction, and maceration. Certain bioactive compounds are heat-sensitive, and the application of conventional processes that involve high temperatures might result to the degradation of these compounds, hence reducing their efficacy and bioavailability. Traditional extraction methods, including maceration or Soxhlet extraction, are ineffective due to their prolonged extraction times, which hinders output [7]. Ultrasound assisted extraction (UAE) is a quick and efficient extraction technique that employs ultrasound to cause rapid movement of solvents by a sequence of compression and rarefaction waves induced in the medium's molecules, resulting in a greater mass transfer rate and faster extraction [8]. In comparison to other novel extraction methods, UAE is economical, convenient due to reduction of extraction time, eco-friendly, cost effective, and adaptable. Researchers favor the usage of ultrasound for the extraction of heat liable compounds from plant materials because of its great repeatability in less time, quicker rate of mass and heat transfer, and reduced instrumental requirements, all of which result in higher product quality. Subsequently, the method favors the use of generally recognized safe solvents due to reduced solvent consumption and lower energy contribution with higher productivity for targeted compounds in a shorter time [9], [10]. Ultrasonication facilitates the disruption of cellular structures such as cell walls and membranes, hence promoting the infiltration of the solvent into the sample. The enhanced penetration facilitates the extraction of target compounds with more efficiency, resulting in a reduced need for solvents compared to traditional extraction techniques such as Soxhlet extraction, maceration, digestion, etc., which utilise larger quantities of organic solvents. UAE is based on the cavitation mechanism, which covers both hydrodynamic and acoustic cavitation processes [11]. The formation, growth, and violent collapse of microbubbles are the three steps of cavitation phenomena that occur at uniform frequency to improve the accessibility of solvent to the solid particles [12], [13]. Recent applications of ultrasonication for the extraction of phytochemicals from agricultural produce were cereal bran [14]; *Ficus auriculata* leaves [15]; guava [16]; pomegranate peel [17].

The process of extraction is a complex non-linear process and needs special mathematical techniques for modeling and optimizing the process. In recent years, artificial neural networks (ANN) have drawn attention to modeling non-linear complex problems due to the ability to predict the data with higher accuracy. The neural network works on the principle of the biological nervous system and it allows to study the effect of process parameters on the responses [18]. Extraction kinetics is very essential for the design of extraction equipment and moreover, the information can be used to accelerate or retard the extraction process based on different conditions and parameters and separation efficiency can be increased [19]. Thermodynamic parameters like enthalpy (ΔH), entropy (ΔS), heat distribution, and Gibb’s free energy (ΔG), can provide information related to understanding the mass and heat transfer rate and thereby controlling it as per requirements [20]. The primary goal of the extraction method is to maximize the extraction of the bioactive compounds while maintaining its quality and integrity. The study of kinetics and thermodynamics during extraction is vital for understanding the underlying type of the process and assessing its practical relevance. Therefore, the goal of the investigation is to analyze and optimize the independent variables of the ultrasound-assisted extraction method for the extraction of phytochemicals from the peels of the persimmon. Additionally, the kinetics and thermodynamics of the UAE approach are examined and analyzed.

## 2 Materials and methods

### 2.1 Raw materials and sample preparation

Persimmon (*Diospyros kaki*) was procured from Malda, West Bengal. The surface of the fruits was cleaned with water to remove dust and dirt. The peels from the fruit were separated by using a stainless-steel knife and peels of the fruit were dried using a freeze dryer (Lyolab Freeze Lab, Lyophilization Systems Inc., USA) for 24 h. The dried peels were ground to fine powder followed by storing the powered peel in an air-tight container at 4˚C.

### 2.2 Chemicals

Ethanol, hexane, gallic acid, 7.5 % $Na_{2}CO_{3}$ solution, 10 % folin–ciocalteu reagent, 2,2-diphenyl-1-picrylhydrazyl (DPPH), 5 % ${NaNO}_{2}$, and 10 % $AlCl_{3}$ were all analytical grade chemicals that were used in the study.

### 2.3 Ultrasound assisted extraction (UAE) of phytochemicals from persimmon fruit peel procedure

The extraction of phytochemicals from the persimmon peel was carried out using ethanol as solvent and by the application of ultrasonication. Apart from being safe for human consumption in small doses, ethanol is characterized by the presence of a polar –OH group and a non-polar -C_2_H_5_ group. This unique composition enables Ethanol to attract both polar and non-polar polyphenol compounds resulting in better extraction yield. Also diluting ethanol with significant amount of water improves the efficiency as polar water molecules increases the polarity of the solution. At first, the freeze-dried persimmon peel powder was mixed with different concentrations of ethanol (40 to 80 %) and various solid to solvent ratios (1:15 to 1:35 g/ml) in a glass beaker. Then the mixed solution was treated with ultrasonication at various power levels (150 to 350 W) using ultrasound with a probe (U500, Takashi, Japan) and different temperatures (30 to 70 °C) for the extraction process. The temperature was continuously monitored with a thermostat and maintained to the desired level by placing the glass beaker in a thermostatic cool water vessel. After ultrasound treatment, the extract was collected and subjected to a centrifugation process in a centrifugal machine at 3600 rpm for 15 min, followed by filtration through Whatman filter paper. The collected supernatant was then quantified for total phenolic content (mg GAE/g d.w.), antioxidant activity (%DPPH inhibition), total beta carotenoid (µg/g d.w.), and total flavonoid content (mg QE/g d.w.).

### 2.4 Modeling of the UAE extraction process

The effect of different process parameters that are ultrasonication power ($X_{U}$), temperature ($X_{T}$), solid to solvent ratio ($X_{S}$) and solvent concentration ($X_{C}$) of ultrasound assisted extraction process on the responses total phenolic content ($Y_{P}$), antioxidant activity ($Y_{A}$), total beta carotenoid ($Y_{B}$) and total flavonoid content ($Y_{F}$) was studied by using an artificial neural network. The experiments were designed by using a central composite circumscribed design with four independent variables which yielded 30 experimental runs and the range of each independent variable with notation was presented in Table 1. The experimental design was modeled using an artificial neural network and the network was formed according to the procedure described by [21]. Briefly, the experimental data was divided into three parts 70 %, 15 %, and 15 % used for training, testing, and validation purpose. The network contains three neurons (independent variables) and four neurons (dependent variables) in the input and output layers respectively. The hidden layer neurons were varied from 3 to 15 and selected based on the training where the network was trained by Levenberg–Marquardt algorithm and log sigmoid was used as the activation function between the layers. The final output parameters of the ANN i.e., weights and bias values were used for investigating the relative influence of process parameters on the response according to the procedure described by [22].

**Table 1:** Evaluated factors, factor notation, and their levels in CCD design.

**Factor**	**Notation**	**Range**		
		**Minimum**	**Average**	**Maximum**
Ultrasonic power, W	$X_{P}$	150	250	350
Temperature, °C	$X_{T}$	30	50	70
Solid to solvent ratio, g/ml	$X_{S}$	1:15	1:25	1:35
Solvent Concentration, %	$X_{C}$	40	60	80

### 2.5 Optimization of the process by genetic algorithm (GA)

The independent variables were optimized by integrating ANN final parameters with the genetic algorithm (GA). The output of the neural network was fed as the initial population of the genetic algorithm. The GA works based on the survival of the fittest by selection, crossover, and mutation. The fitness function was fed to the algorithm with the maximization of the responses and presented in equation Eq. (1). The best combination was optimized by fitness value.

(1) $$\text{FF}=\left\{\begin{array}{c} {\text{maxY}}_{\text{P}}\left({\text{X}}_{\text{U}},{\text{X}}_{\text{T}},{\text{X}}_{\text{S}},{\text{X}}_{\text{C}}\right)\\ {\text{maxY}}_{\text{A}}\left({\text{X}}_{\text{U}},{\text{X}}_{\text{T}},{\text{X}}_{\text{S}},{\text{X}}_{\text{C}}\right)\\ {\text{minY}}_{\text{B}}\left({\text{X}}_{\text{U}},{\text{X}}_{\text{T}},{\text{X}}_{\text{S}},{\text{X}}_{\text{C}}\right)\\ {\text{minY}}_{\text{F}}\left({\text{X}}_{\text{U}},{\text{X}}_{\text{T}},{\text{X}}_{\text{S}},{\text{X}}_{\text{C}}\right)\\ 150⩽{\text{X}}_{\text{U}}\left(W\right)⩽350\\ 30⩽{\text{X}}_{\text{T}}\left({}^{\circ}\text{C}\right)⩽70\\ 1:15⩽{\text{X}}_{\text{S}}\left(g/ml\right)⩽1:35\\ 40⩽{\text{X}}_{\text{C}}\left(\%\right)⩽80 \end{array}\right)$$

### 2.6 Quantification of phytochemicals from UAE persimmon peel extract

#### 2.6.1 Quantification of total phenolic content (TPC)

The folin-Ciocalteu method was used in the quantification of the total polyphenol content (TPC) of UAE extract from the persimmon peel [23]. In brief, 0.5 ml of ethanolic persimmon peel extract was added to 2.5 ml 10 % FC reagent and left for 5 min followed by adding 2.5 ml of 7.5 % $Na_{2}CO_{3}$. Then the mixture was mixed well and allowed to incubate in the dark at room temperature for 30 min and in the absence of peel extract, the blank was prepared. Using a spectrophotometer, the absorbance resulted at $\lambda_{\max}=765nm$ after the incubation. For the determination of total phenolic content each experiment was conducted in and the mean absorbance data was recorded. The same procedure was repeated for the standard solution of gallic acid, the calibration line was construed and the total phenolic content of persimmon fruit peel was calculated using the equation presented in Eq. (2).

Total phenolic content ${(Y}_{P})=\frac{c\;\times \;V}{w}$ (2)

Where c indicates the standard concentration (gallic acid), v indicates UAE persimmon fruit peel extract volume, and w weight of the persimmon peel powder.

#### 2.6.2 Estimation of antioxidant activity of persimmon fruit peel

The antioxidant capacity of UAE persimmon peel extract was estimated in terms of %DPPH inhibition and followed the process described by Alara (2018) [24]. Concisely, 0.1 mM DPPH solution was prepared using 0.004 g of DPPH crystalline solid mixed with 100 ml of analytical grade ethanol of purity 99.95 % and kept at 4˚C. The UAE persimmon peel extract of volume 0.2 ml is mixed with 2 ml of ethanol and 2 ml of DPPH solution. The mixture was kept for 30 min incubation at room temperature in a dark place. The absorbance of the mixture after incubation was recorded at 517 nm against a blank (ethanol) using UV–visible spectrophotometer. The antioxidant activity was estimated by the equation presented in Eq. (3).

Antioxidant Capacity ($Y_{A}$) = $\left(1-\frac{A_{S}}{A_{C}}\right)\times 100$ (3)

where $A_{c}$ indicates the absorbance of the mixture of DPPH solution and ethanol, $A_{s}$ indicates the absorbance of the solution mixture containing the sample.

#### 2.6.3 Estimation of total β-carotenoid content

The spectrophotometry method was used for the determination of the total β-carotene content of the UAE persimmon peel extract according to the procedure described by Machmudah & Goto (2013) [25]. First UAE extract was mixed with acetone and filtered through Whatman’s no 1 filter paper. Then, β-carotene was extracted by pouring the extract with hexane in separatory funnel followed by further dilution of extract at 1:50 ratio with hexane. After discarding the watery phase, absorbance (A) of the resultant solution was measured at 450 nm with pure hexane employed as blank. Total β-carotene content was expressed in mg/100 g d.w. and was calculated using Eq. (4).

(4) $$Total\;\beta -carotene\;\left(Y_{B}\right)=\frac{A\times D}{A^{1\%}\;}\times C^{1\%}$$

where A indicates the absorbance at 450 nm; D indicates the dilution factor; $A^{1\%}$ indicates the absorption coefficient, 2592 AU and $C^{1\%}$ indicates the concentration of 1 % standard β-carotene solution, 10 mg/ml.

#### 2.6.4 Total flavonoid content

The total flavonoid content of UAE persimmon peel extract was quantified using the spectrophotometry method according to the process described by Zeng et al., 2019 with slight modifications [26]. Briefly, UAE persimmon peel extract of 0.5 ml was mixed with 0.1 ml of 5 % ${NaNO}_{2}$ and incubated for 5 min, thereafter 0.1 ml of 10 % $AlCl_{3}$ was added to the mixture. The mixture was mixed well for 1 min and then 1 ml of NaOH was added quickly. The mixture absorbance was measured at 510 nm using UV-Spectrophotometer. For the calibration curve different concentrations of quercetin (QE) standard solution was prepared and a similar procedure was followed for mixing and measuring the absorbance. The total flavonoid content of persimmon peel extract was expressed in mg of QE/g persimmon peel dry weight.

### 2.7 Kinetic modeling of responses $Y_{P}$, $Y_{A}$, $Y_{B}$ and $Y_{F}$ from UAE persimmon peel extract

The pseudo kinetic second order kinetic model was implemented for studying the extraction kinetics of extract obtained from persimmon fruit peel by the application of ultrasonication. The pseudo second order kinetic model was used for the investigation of the extraction kinetics of bioactive compounds from plant materials by various authors [27], [28]. In the current investigation, the extraction was executed for an extraction time of 25 min by changing temperature in the range of 30–––70 °C, and the other process parameters were kept at the optimum condition obtained by the hybrid ANN-GA model. The obtained observed data was fitted with pseudo second order kinetic model presented in Eq. (7) and Eq. (8).

(7) $$\frac{dC_{t}}{\mathrm{dt}}=k{\left(C_{s}-C_{t}\right)}^{2}$$

(8) $$C_{t}=\frac{C_{s}^{2}kt}{1\;+{\;C}_{s}kt}$$

where $C_{t}$ indicate the specific phytochemical concentration in the UAE extract at a particular extraction time t, $C_{s}$ indicates the saturated phytochemical concentration in the UAE extract and k indicated the kinetic model rate constant.

The activation energy for each response was calculated by correlating the pseudo second order kinetic rate constant (k) with temperature. The Arrhenius equation was used for the determination and presented in Eq. (9)

(9) $$k=k_{0}exp\left(-\frac{E_{a}}{\mathrm{RT}}\right)$$

where $E_{a}$ is the activation energy, R is the gas constant (8.314 J/mol K).

### 2.8 Thermodynamics of UAE

The major regulating phase of extraction in the solid solvent extraction method was the diffusion of the targeted compound out of the solid matrix into the solvent. Gibb's free energy computes the useful work accessible from a thermodynamic system at constant temperature and pressure. The enthalpy and the entropy of each phytochemical compound in the UAE extract help in calculating Gibb's energies throughout chemical changes. The equation representing Gibb's free energy ($\Delta G^{{}^{\circ}}$), entropy ($\Delta S^{{}^{\circ}}$), and enthalpy ($\Delta H^{{}^{\circ}}$) was presented in Eq. (10).

(10) $$\Delta G^{{}^{\circ}}=\Delta H^{{}^{\circ}}-T\Delta S^{{}^{\circ}}$$

The isotherm equation for $\Delta G^{{}^{\circ}}$ was shown in Eq. (11)

(11) $$\Delta G^{{}^{\circ}}=-RTlnK_{\mathrm{eq}}$$

The entropy ($\Delta S^{{}^{\circ}}$) and enthalpy ($\Delta H^{{}^{\circ}}$) of the extraction were estimated by the Van’t Hoff equation shown in Eq. (12).

(12) $$\ln K_{e}=-\frac{\Delta H^{{}^{\circ}}}{\mathrm{RT}}+\frac{\Delta S^{{}^{\circ}}}{R}$$

where ‘$K_{e}$’ indicates the equilibrium constant for extraction of the specific phytocompound and is determined using the equation represented in Eq. (13).

(13) $$K_{e}=\frac{C_{s}}{C_{max}-C_{s}}$$

where $C_{s}$ indicates the concentration of response in the UAE extract after an extraction time of 25 min; T indicates the temperature used for extraction K and $C_{\max}$ indicates the concentration response extracted after a complete extraction using the optimized solvent concentration.

### 2.9 Statistical analysis

The statistical tools implemented for checking the accuracy of the ANN, GA, and kinetic model for the UAE extraction process were presented in Eq. (14), (15), (16), (17) [29].

(14) $$R^{2}=1-\frac{\sum_{i=1}^{n}\left(Y_{p}-Y_{e}\right)}{\sum_{i=1}^{n}\left(Y_{a}-Y_{e}\right)}$$

(15) $$RMSE=\sqrt{\frac{\sum_{i=1}^{n}\left(Y_{p}-Y_{e}\right)}{n-1}}$$

(16) $$\chi^{2}=\frac{\sum_{i=1}^{n}{\left(Y_{p}-Y_{e}\right)}^{2}}{Y_{e}}$$

(17) $$R_{d}=\frac{100}{n-1}\sum_{i=1}^{n}\frac{\left|{Y_{e}-Y}_{p}\right|}{Y_{e}}\;$$

where $Y_{p}$ indicates the expected value from the model, $Y_{e}$ indicate experimental value, $Y_{a}$ is the mean value, and n indicates the total experimental runs.

## 3 Results and discussion

### 3.1 Modeling and optimization of the UAE extraction process

#### 3.1.1 Artificial neural network modeling of UAE extraction of phytochemicals from PFP

**Fig 1:**
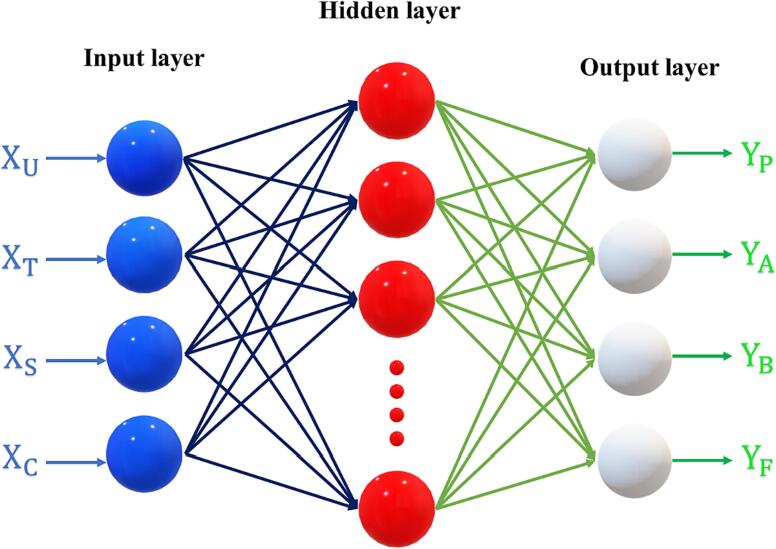
Proposed ANN architecture for ultrasound assisted extraction of phytochemicals from persimmon peel.

The hidden neurons in the hidden layer were found to be 12 neurons according to the lower error value of the network during training of the network. The ANN was trained using the Levenberg algorithm and the best structure had the lowest relative deviation of less than 0.001. The best network architecture contained 3 input neurons, 4 output neurons, and 12 hidden neurons in the input layer, output layer, and hidden layer respectively illustrated in Fig. 1. The ANN parameters (u, w, Th, and To) final values after completion of 5000 cycles were presented in Table 2. The obtained parameters of u, w, Th, and To were applied for predicting the dependent variables using independent variables. The designed ANN model has a higher $R^{2}$ and lower error values which show the successful implementation of ANN modeling in predicting the experimental data. The effect of the different independent variables on responses, i.e., total phenolic content, antioxidant activity, total beta carotenoid, and total flavonoid content was analyzed by ANN model according to the obtained parameters and were represented in Table 3.

**Table 2:** Artificial neural network weights and bias values (u, w, T_h_, T_w_).

8.11	2.98	4.35	−0.08	6.15	−3.08	4.64	1.67	2.82	−7.44	13.95	1.69
−4.77	−0.41	−1.49	7.15	−1.24	−2.70	1.07	1.39	8.09	6.46	3.58	4.45
0.53	1.19	−3.80	4.81	3.62	5.60	6.54	1.38	−0.75	−0.90	−0.17	12.64
−7.44	6.17	2.07	−2.74	−1.14	5.65	3.66	−2.92	1.31	0.75	0.55	1.71

2.82	0.33	4.65	2.47
−5.69	1.22	−3.58	5.39
−3.16	3.93	1.57	2.49
3.81	4.21	4.38	3.56
7.31	−6.14	−8.06	7.90
1.53	−3.35	0.10	−0.35
−1.41	2.71	3.44	0.86
−6.30	−3.23	−5.29	−5.53
−0.78	−1.84	−6.06	5.89
−0.69	−0.03	1.57	−8.06
3.49	0.32	4.89	−6.89
−2.29	1.38	−2.76	−3.70

−4.73
−5.77
−2.44
2.96
−5.44
−1.43
−3.11
−1.19
4.10
−1.61
0.79
3.98

−1.26
−2.46
1.00
0.54

**Table 3:** Relative influence of the coded values of independent variables on the response.

$x_{P}$	$x_{T}$	$x_{S}$	$x_{C}$	$\Delta y_{P}$	$R_{d}$	$\Delta y_{A}$	$R_{d}$	$\Delta y_{B}$	$R_{d}$	$\Delta y_{F}$	$R_{d}$
+1	0	0	0	0.412	0.469	0.619	0.276	0.425	0.338	0.968	0.297
−1	0	0	0								
0	+1	0	0	−0.319		0.504		−0.176		−0.581	
0	−1	0	0								
0	0	+1	0	−0.249		−0.203		0.253		−0.284	
0	0	−1	0								
0	0	0	+1	−0.032		0.327		−0.244		0.395	
0	0	0	−1								

#### 3.1.2 Effect of process parameters on the TPC

**Fig. 2:**
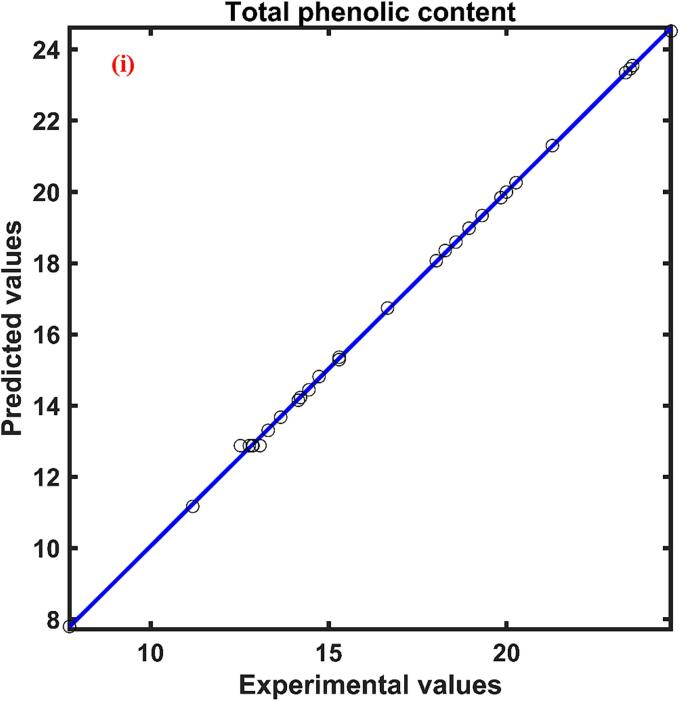

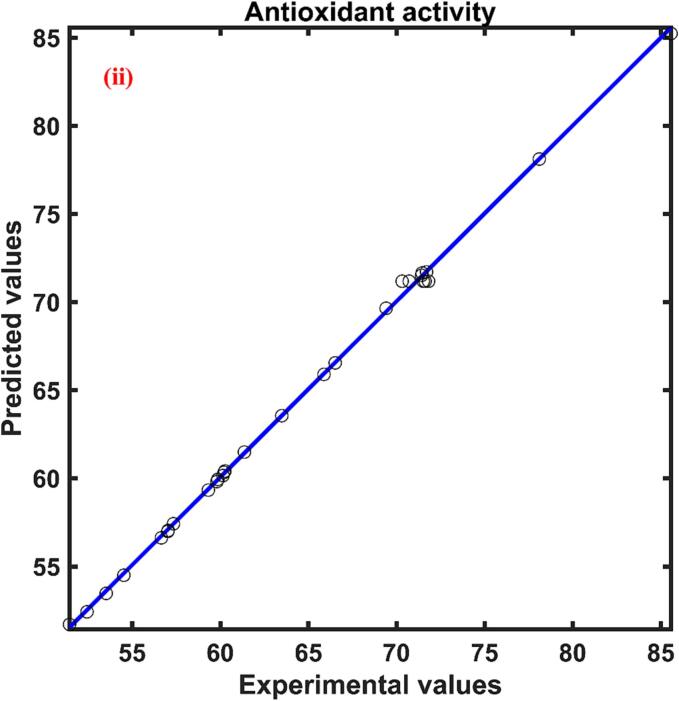

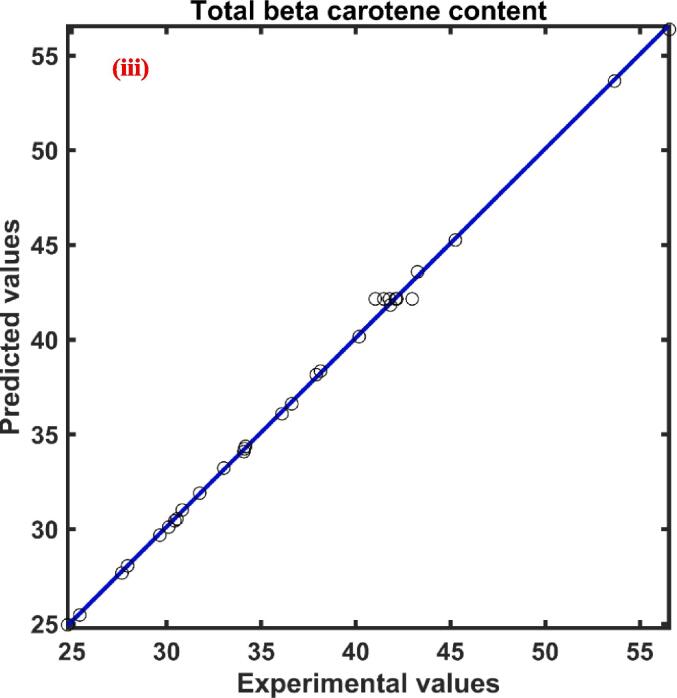

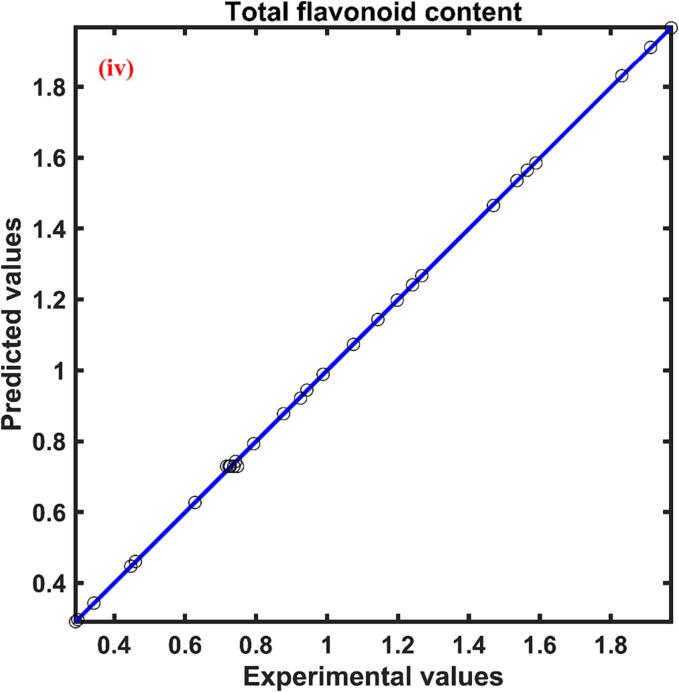
Experimental and ANN predicted plot for (i) Total phenolic content, mg GAE/g d.w.; (ii) Antioxidant activity, %DPPH inhibition; (iii) Total beta carotenoid content, µg/g d.w.; and (iv) Total flavonoid content mg QE/g d.w. of UAE persimmon peel extract.

The experimental range of total phenolic content of the persimmon peel extract with respect to the experimental design was found to be 7.723–––24.619 mg GAE/g d.w. The experimental total phenolic content data and ANN model predicted data are compared and illustrated in Fig. 2(i) Excellent correlation was found between experimental and predicted data which was evident by the correlation coefficient close to unity. The relative effect of process variables on the total phenolic content was presented in Table 3. Out of the four process parameters, ultrasonication power (0.412) was found to have a positive influence on the $Y_{PC}$ of the persimmon peel followed by temperature (-0.319), solvent solid ratio (-0.249), and solvent concentration (-0.032). Positive symbol signifies that increase in the process parameter improved $Y_{PC}$ while negative sign implies the reverse effect.

The combination of solvent extraction with ultrasonication during the extraction of phenolic compounds from the plant materials has the advantage of an acoustic cavitation mechanism that helps in higher extraction yield into the solvent. The ultrasound waves in the solvent create high shear forces that have the ability to disrupt the walls of the cell and allow the diffusion of the solvent into the cells and help in the dissolving of the targeted substance [30]. The results were in agreement with the effect of ultrasonication power on the total phenolic content of the extract obtained from *Cassia auriculata* leaves by ultrasound assisted extraction method [31]. The rise in temperature decreases the solvent viscosity, which results in an increase in vapor pressure and helps in the development of extra bubbles during ultrasonication. But, the pressure difference between the inner and outer sides of the bubbles was lesser and the bubbles when reaches an unstable state collapsed with lesser intensity. This attributes to the negative influence of higher temperatures when combined with ultrasound [9]. The drop in total phenolic content at a higher solid to solvent ratio can be due to the leaching of other compounds like proteins and polysaccharides into the solvent during extraction which in turn decreased the dissolution of phenolic composites [32]. Comparable results of a decrease in total phenolic content of UAE mango peel extract were reported with an increase of solid solvent ratio of 1:30 to 1:40 mg/ml during ultrasound assisted extraction of phytochemicals from mango peel [33]. Mixing both water and ethanol has the advantage of dissolving polar as well as less polar phenolic compounds effectively [34]. The effectiveness of extracting polyphenols is dependent upon several factors, including the dielectric constant, the characteristics of the phenolic compounds, the structure, the correlation with the solvent's polarity, and the degree of polymerization [35]. Similar trends of decrease in total phenolic content with the rise of solvent concentration were described during the extraction of phenolics from *Terminalia chebula Retz.* fruit by the application of ultrasonication [36]. The total phenolic content was approximately 350 mg GAE/g d.w. when extracted with 60 % ethanol. However, increasing the ethanol concentration to 100 % resulted in a decrease of approximately 37 % in total phenolic content.

#### 3.1.3 Influence of independent variables on antioxidant activity of UAE persimmon peel extract

The highest antioxidant activity ($Y_{A}$) value of the UAE persimmon peel extract was found to be 85.584 % DPPH inhibition and the lowest value was approximately 40 % less than that. The ANN model was found to forecast the antioxidant activity of persimmon peel extract at different combinations of experimental conditions indicated by the higher correlation coefficient and the predicted antioxidant activity range was found to be 51.717–––85.237 % DPPH inhibition. The ANN predicted and experimental antioxidant data were plotted against each other and shown in Fig. 2(ii) The influence of independent parameters on the antioxidant activity of the persimmon peel extract was presented in Table 3 where the independent variable $X_{U}$ (0.619), $X_{T}$ (0.504) and $X_{C}$ (0.327) had a positive influence on the antioxidant activity of the extract evidenced by the positive sign. Whereas the fourth variable $X_{S}$ (-0.203) found to have a negative influence evidenced by the negative sign presented in Table 3. According to the value of the relative influence of the process variables, $X_{U}$ found to have a higher influence when compared with the $X_{T}$, $X_{S}$ and $X_{C}$ parameters. The solvent solid ratio was found to have a lower influence on the antioxidant activity.

The enhanced antioxidant activity in the extract with the increase of ultrasonication power can be attributed to the high-speed jets of liquids formed in the solvent due to the collapse of the bubbles that lead into the surface and generates shockwave damage to the cell of the plant material [37]. The positive influence of solvent concentration on the antioxidant activity was also reported for the ultrasound assisted extraction of bioactive compound from lime peel [38]. The decrease in antioxidant activity with an increase in solid liquid ratio may be attributed to the saturation phase of the extraction of phytochemicals into the extraction and further exposure to temperatures might have degraded the heat sensible compounds in the extract [39]. On studying the effect of blanching and drying methods on the persimmon peel, application of heat significantly degraded the phenolics, β-carotene content which may attributed to a maximum 40.94 % loss in antioxidant activity [40].

#### 3.1.4 Effect of process parameters on the total beta carotenoid content

The maximum and minimum values of total beta carotenoid content ($Y_{B}$) for the UAE persimmon peel extract were found to be 24.782–––56.558 µg/g d.w. The ANN predicted $Y_{BC}$ data versus experimental $Y_{BC}$ data was presented in Fig. 2(iii) The regression coefficient value was found to be 0.99 which signifies the good relationship between the observed and ANN predicted data for the total beta carotenoid content of persimmon peel extract. The effect of four independent variables on the extraction of beta carotenoid found in the order $X_{U}\left(0.425\right)>X_{S}\left(0.253\right)>X_{C}\left(0.244\right)>X_{T}\left(0.176\right)$ as indicated by the magnitude of the relative effect calculated by ANN model presented in Table 3. The study of relative influence also revealed that $X_{U}$ and $X_{S}$ had a positive influence while the $X_{C}$ and $X_{T}$ had a negative effect on the total beta carotenoid content of the extract.

The improved extraction of beta carotenoid from the persimmon peel with the rise of ultrasonication power might be attributed to the disruption of cell walls and vacuoles of plant tissues. A comparable effect of ultrasonication on total beta carotenoid was reported during beta carotenoid extraction from carrot residue during ultrasound assisted extraction with ethanol as solvent where the total beta carotenoid was increased to around 72 % when ultrasound power was increased from 20 to 100 W [41]. The negative influence of temperature related to the extraction yield of total beta carotenoid from persimmon peel might be due to the degradation at higher temperatures. The results of a decrease in total beta carotenoid in the extract at higher temperatures during ultrasonication were also reported for the extraction of carotenoids from oil palm fronds during ultrasound assisted extraction method [42]. The positive influence of the ultrasonication power and solid solvent ratio on the yield of beta carotenoid from the persimmon peel into the extract during UAE might be attributed to the larger concentration gradient during the diffusion from the solid into the solution at a higher solid to solvent ratio. A similar effect of solvent to solid ratio was reported for carotenoid content in the extract obtained from pomegranate wastes by the application of ultrasonication during extraction [43].

#### 3.1.5 Effect of process parameters on the total flavonoid content

The total flavonoid content obtained for the UAE persimmon peel extract was found to be in the range of 0.291–––1.970 mg QE/g at different experimental conditions. The neural network predicted the observed values with good agreement which can be evident by the correlation coefficient close to unity. The plot between the experimental and the ANN forecast data were shown in Fig. 2(iv). The influence of independent parameters on the total flavonoid content was presented in Table 3. The relative effect specified that the ultrasonication power (0.968) had a higher impact on the extraction of flavonoids from the persimmon peel into the solvent during ultrasound assisted extraction and solid liquid ratio (-0.248) had a lower impact when compared with other process parameters. The sign of each parameter presented in Table 3 unveils the positive effect of ultrasonication power and solvent concentration on the total flavonoid content and the negative influence on the response by solid liquid ratio and temperature.

Similar results were reported where the total flavonoid content of kiwi fruit without ultrasonication was 26.60 mg CE/100 ml and the flavonoid content was enhanced to 54.68 mg CE/100 ml with the application of 400 W ultrasonication power and 20 kHz of frequency for 16 min [44]. The influence of temperature on the flavonoid content was similar to the phenolics where the higher temperatures were less prominent in the extraction of flavonoids from the persimmon peel during ultrasound assisted extraction. The effect of temperature on flavonoids during ultrasonication (100 W power and 12 min extraction) were close to the results reported for the cumin seed where the increase of temperature from 25 to 35 °C had a positive influence while the further rise of temperature from 35 °C to 65 °C had a negative influence on the catechin content of the extract [45]. The negative influence of solid to solvent ratio on the flavonoid content could be credited to the increase in the surface tension and viscosity of the solvent that results in a lower effect of cavitation [46]. Comparable trends of ethanol as solvent were reported where an increase of concentration to 50 % improved the yield of flavonoids from the peels of the mango but a further increase of concertation to 100 % slightly decreased the flavonoid content in the extract during extraction of bioactive compounds from peels of the mango by the application of ultrasound [47].

#### 3.1.6 Optimization by genetic algorithm

By applying genetic algorithm, the optimum values were calculated according to the fitness function equation presented in Eq. The iteration was done in such a manner that all four responses were to be maximized. The integrated ANN-GA model formed 18 sets of solutions in which the highest set having fitness value (3.087) was selected as the optimum condition according to the rule set for the response. The optimized condition for the process parameters ultrasonication power, temperature, solid liquid ratio, and solvent concentration according to the model was found to be 230.176 W, 50.661 °C, 28.273 ml/g, and 62.750 % respectively during UAE extraction of persimmon peel. The predicted value for the response total phenolic content (mg GAE/g d.w.), antioxidant activity (% DPPH inhibition), total beta carotenoid (µg/g d.w.), and total flavonoid content (mg QE/g) at optimum condition by ANN-GA was found to be 17.860, 70.643, 54.281 and 1.962 respectively. The optimum condition was validated by comparing the result, the experiments were conducted at the optimum condition and compared with the predicted value. The experimental value for the responses in the same sequence was found to be 16.698 ± 0.142 mg GAE/g d.w., 69.039 ± 0.994 % DPPH inhibition, 49.947 ± 0.889 µg/g d.w. and 1.817 ± 0.032 mg QE/g d.w. The experimental values were close to the predicted values of the ANN-GA model which was evident by the statistical parameter relative deviation of less than 10 %, shown in Table 4.

**Table 4:** Relative deviation between experimental and predicted values obtained at optimal condition.

**Responses**	**Experimental values**	**Predicted values**	**Relative deviation (%)**
$Y_{T}$**(mg GAE/g d.w.)**	16.698 ± 0.142	17.860	6.960
$Y_{A}$**(%DPPH inhibition)**	69.039 ± 0.994	70.643	2.323
$Y_{B}$**(µg/g d.w.)**	49.947 ± 0.889	54.281	8.678
$Y_{F}$**(mg QE/g d.w.)**	1.817 ± 0.032	1.962	7.988

### 3.2 Kinetic modeling

#### 3.2.1 Extraction of phenolic compounds

**Fig. 3:**
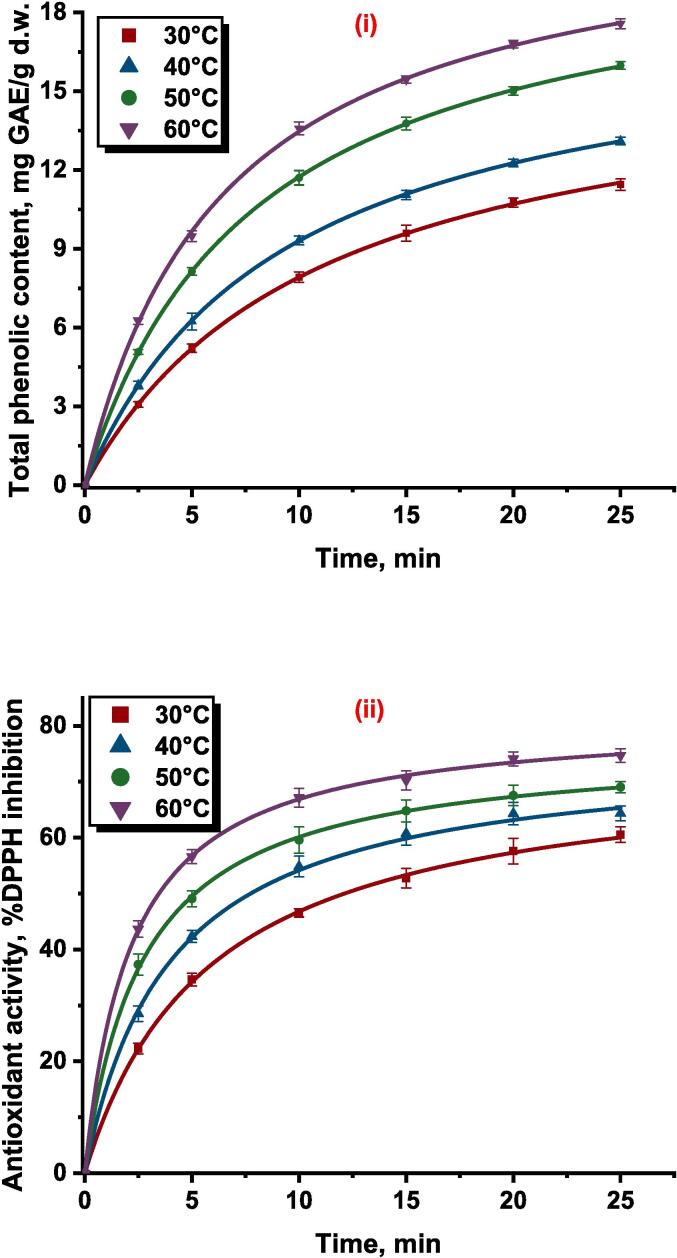

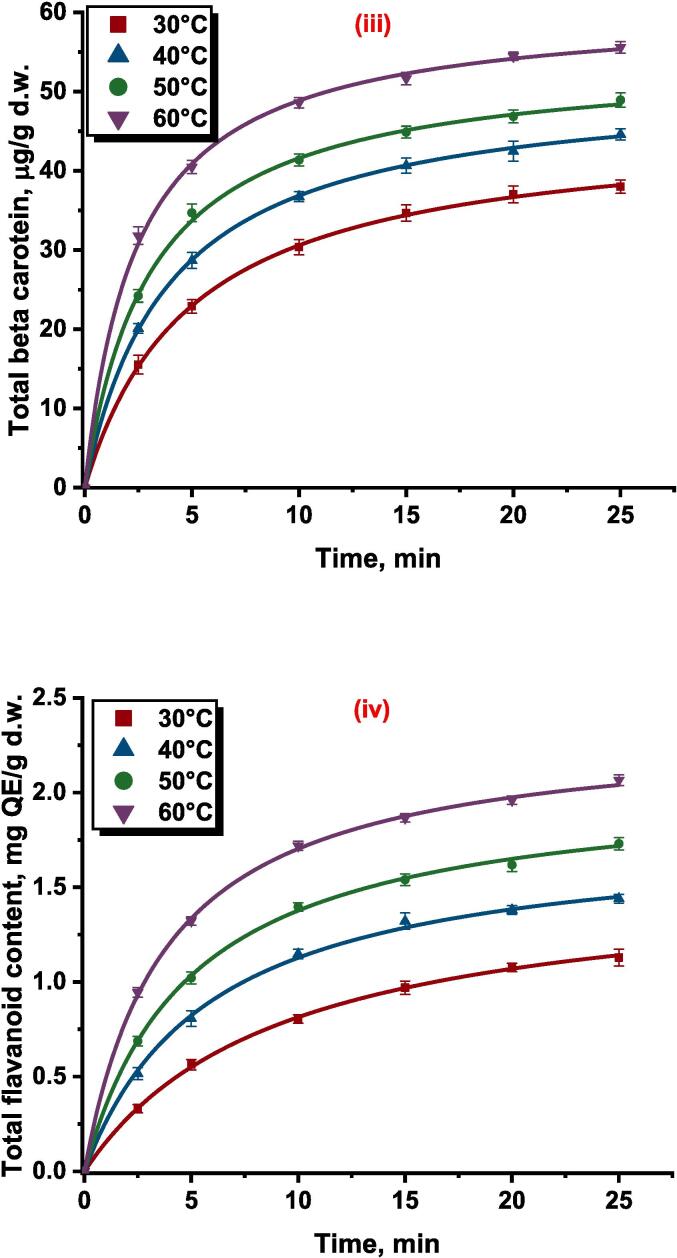
Pseudo second order kinetic model fitting for (i) Total phenolic content, mg GAE/g d.w.; (ii) Antioxidant activity, %DPPH inhibition; (iii) Total beta carotenoid content, µg/g d.w.; and (iv) Total flavonoid content mg QE/g d.w. of UAE persimmon peel extract.

The total phenolic content of UAE extract obtained from persimmon peel after 25 min of extraction was found to be in the range of 11.446 ± 0.217 – 17.571 ± 0.192 mg GAE/g d.w. by changing 30 °C temperature to a temperature of 60 °C. The $Y_{P}$ was increased with the rise of temperature, and at particular temperatures, $Y_{P}$ was enhanced with a step up in extraction time, illustrated in Fig. 3(i). At a temperature of 50 °C, increase of extraction time from 5 to 15 min the $Y_{P}$ yield was increased by approximately 69 % while the further increase of extraction time 15 to 25 min increased 16 % of $Y_{P}$ yield. Therefore, it can be observed that the $Y_{P}$ yield was higher at the initial phase of extraction and negligible raise was observed at the final stage of extraction. A similar pattern of higher $Y_{P}$ yield at the initial phase of extraction and lower extraction yield of phenolic compounds into the extract was found for other extraction temperatures. Comparable results of the faster release of phenolics from olive leaves into the solvent at the initial stage of extraction were described during extraction by the application of ultrasonication [48].

The kinetic model for the extraction of the response $Y_{P}$ at different temperatures was found to fit the experimental data with $R^{2}$ higher than 0.961, $\chi^{2}$ and RMSE lower than 0.889 and 0.790 respectively. The parameters of the statistical tool signify that the model was found to predict the data with higher accuracy and lower error, presented in Table 5. The experimental data along with the model predicted values were plotted and presented in Fig. 3(i) The kinetic model parameters rate constant (k) and saturation concentration ($C_{s}$) was found to be in the range of 0.556 $\times 10^{-2}$ – 0.702$\times 10^{-2}$ g d.w./mg GAE min and 16.536 – 22.136 mg GAE/g d.w. respectively, shown in Table 5. Both the parameters of the pseudo kinetic model were observed to increase with the rise of temperature. The increase in the rate constant for the $Y_{P}$ yield with an increase in temperature might be due to the necessary of huge thermal energy for the penetration of solute as well as the higher temperature reduced the viscosity of the solvent helped in the rapid speed of solvent into the cell matrix of the plant [49].

**Table 5:** Parameters of second order kinetic model for responses at different temperatures.

**Responses**	**Temperature, °C**	$k\times 10^{-2}$	$C_{s}$	$R^{2}$	$\chi^{2}$	**RMSE**
$Y_{T}$	30	0.556 ± 0.011	16.536 ± 0.101	0.944	0.081	0.285
	40	0.597 ± 0.007	17.976 ± 0.055	0.976	0.010	0.101
	50	0.607 ± 0.004	20.968 ± 0.045	0.955	0.007	0.083
	60	0.702 ± 0.019	22.136 ± 0.154	0.978	0.028	0.166
$Y_{A}$	30	0.233 ± 0.008	74.006 ± 0.691	0.986	0.006	0.076
	40	0.332 ± 0.019	75.740 ± 0.998	0.979	0.003	0.059
	50	0.479 ± 0.013	76.519 ± 0.347	0.963	0.006	0.077
	60	0.564 ± 0.018	81.462 ± 0.432	0.959	0.003	0.053
$Y_{B}$	30	0.439 ± 0.014	45.816 ± 0.344	0.961	0.013	0.113
	40	0.497 ± 0.010	51.325 ± 0.242	0.983	0.025	0.158
	50	0.603 ± 0.029	54.314 ± 0.496	0.972	0.790	0.889
	60	0.678 ± 0.036	60.749 ± 0.483	0.965	0.016	0.128
$Y_{F}$	30	7.007 ± 0.333	1.560 ± 0.022	0.948	0.007	0.083
	40	9.518 ± 0.651	1.790 ± 0.028	0.975	0.004	0.059
	50	9.824 ± 0.525	2.058 ± 0.027	0.959	0.019	0.136
	60	11.215 ± 0.471	2.350 ± 0.021	0.990	0.007	0.085

Similar findings were reported where an increase of temperature from 35 to 85 °C increased the rate constant (g d. w. /mg GAE min) of pseudo second order kinetic model from 0.099 to 0.107 during extraction of the phenolic compound from leaves of jamun using convention extraction method at a ratio of solvent to solid of 35 ml/g [50]. Comparable trends of kinetic model parameters with change in temperature were reported during the extraction of phenolic compounds from chamomile flowers powder by conventional extraction method with water as a solvent, where the rate constant and equilibrium concentrations values increased from 0.028 to 0.31 ${min}^{-1}$ and 14.6 to 20.2 mg GAE/g with a rise of temperature from 57 °C to 80 °C respectively [51].

#### 3.2.2 Extraction kinetics of response antioxidant activity

The antioxidant activity of persimmon fruit peel extract was expressed in terms of % DPPH inhibition. After 25 min of UAE extraction, the highest antioxidant activity of 74.695 % was observed for the extract obtained at an extraction temperature of 60 °C whereas the lowest antioxidant activity was observed for the extract obtained at a temperature of 30 °C which was 18.914 % lower than the highest antioxidant activity. The results signify that the antioxidant activity was increased with the increase in temperature, which can be noticed in Fig. 3(ii). The enhancement of the antioxidant activity of extract containing bioactive compounds with an increase of heat might be due to probable acceleration of diffusion rate, increased phenolic solubility, reduction in solvent viscosity, higher extraction yield, increased efficiency of mass transfer, and reduction in surface tension phenomena [52]. The results of the antioxidant activity of persimmon peel versus time at various temperatures were plotted and presented in Fig. 3(ii). From this figure, it can be visualized that the antioxidant activity of the extract increased sharply at the beginning stage of extraction and the activity declined at the end of the process. The increase in antioxidant activity at the initial phase of extraction due to the washing stage and slow extraction at the final stage attributed to attaining equilibrium and internal diffusion phenomena [53]. Comparable trends of positive influence of temperature on antioxidant activity were reported during the extraction of bioactive compounds from *Baccharis dracunculifolia* [54].

The antioxidant activity data with respect to time at various extraction temperatures during UAE of bioactive compounds from persimmon peel was predicted adequately by the kinetic model which was evident by the statistical parameters presented in Table 5. The calculated statistical parameters $R^{2}$, $\chi^{2}$ and RMSE for the kinetic model when fitted with antioxidant activity data of the persimmon peel extract at temperatures 30, 40, 50, and 60 °C was found to be 0.986, 0.979, 0.963, and 0.959; 0.006, 0.003, 0.006 and 0.003; and 0.076, 0.059, 0.077, and 0.053 respectively. The kinetic model parameters of antioxidant activity of UAE persimmon peel extract followed a similar trend of kinetic parameters of total phenolic content where both the parameters of the kinetic model were observed to increase with the rise of temperature. Kinetic model parameters, rate constant (k), and saturation concentration ($C_{s}$) was found to be in the range of 0.233 $\times 10^{-2}$ – 0.564 $\times 10^{-2}$ ${min}^{-1}$ and 74.006 – 81.462 % DPPH inhibition respectively, shown in Table 5.

#### 3.2.3 Extraction kinetics of total beta carotenoid content

Post varying temperature from 30 to 60 °C for an extraction time period of 25 min it was found that the total beta carotenoid content (TBC) of UAE extract obtained from persimmon peel was a minimum of 38.011 ± 0.838 µg/g d.w. and a maximum of 55.596 ± 0.719 µg/g d.w. With the rise in temperature, the TBC was also observed to increase, and at particular temperatures, with an increase in extraction time, the TBC also increased, illustrated in Fig. 3(iii). The figure shows that the initial phase of extraction yield of beta carotenoid was higher as compared to the final stage of extraction. A similar pattern of TBC yield was observed in the case of other extraction temperatures. The rise of the temperature during extraction might have enhanced the rates of diffusion, the solubility of the targeted compound, the transfer of compounds, and decreased the viscosity and surface tension of the solvents, permitting a quicker extraction process with less usage of solvent [55].

The average value of statistical parameters $R^{2}$, $\chi^{2}$ and RMSE was observed to be 0.963, 0.032, and 0.159 respectively revealing that there was a good correlation between the observed values and the predicted values of the pseudo second order kinetic model for the extraction of total beta carotenoid at different temperature conditions. The experimental data along with the model predicted values were presented in Fig. 3(iii). The kinetic model parameters rate constant (k) and saturation concentration ($C_{s}$) was found to be in the range of 0.439 $\times 10^{-2}$ – 0.678$\;\times 10^{-2}$ g d.w./ µg min and 45.816 – 60.749 µg/g d.w. respectively, shown in Table 5. An increase in temperature affected both the parameters of the model in a positive way. Similar findings were reported where an increase in temperature from 30 to 45˚C increased the $C_{s}$ value from 1071.811 to 1219.512 µg carotenoids/100 g of d.w. during extraction of TBC from passion fruit peel using the UAE method [56].

#### 3.2.4 Extraction kinetics of total flavonoid content

Flavonoid compounds are secondary metabolites of agricultural produce that hold an aromatic ring bearing at least one hydroxyl group [57]. The process variables temperature and time were found to have a positive influence on the extraction of flavonoids from the persimmon peel by the application of ultrasonication. The increase of temperature improved the extraction of flavonoids which signifies that the temperature range selected for the extraction had no adverse effect on the heat liable flavonoid compounds. Similar to the temperature the 25 min extraction time was sufficient to extract flavonoids from the matrix of the sample without being prone to degradation. The maximum total flavonoid content of 2.066 ± 0.028 mg QE/g was found for the persimmon peel extracted at a temperature of 60 °C and 25 min of extraction time. The total flavonoid of UAE extract obtained at temperatures of 50, 40, and 30 °C for 25 min extraction time was found to decrease by approximately 16 %, 30 %, and 45 % when compared with the maximum total flavonoid content. At a specific temperature of 40 °C, the total flavonoid content was found to increase rapidly with the increase of extraction time to 15 min but thereafter the increase was negligible which can be visualized in Fig. 3(iv). A comparable trend of flavonoid extraction was observed with respect to extraction time for the UAE persimmon peel extract for other extraction temperatures. The increase in the flavonoid content in the extract with an increase in extraction temperature might be associated with softening of the tissue and weakening of the phenol–protein and phenol-polysaccharide interactions which causes more polyphenols to migrate in the solvent as flavonoid compounds are also usually found as glycosides [58], [59]. Identical results were reported where the total flavonoid content in the extract increased from 38 mg QE/100 g to 81 mg QE/100 g, with the rise of temperature from 25 to 60 °C but further increase in temperature to 70 °C having an adverse effect on the yield of flavonoids during ultrasound assisted extraction of phenolics from peach fruit [60].

The pseudo second order kinetic equation was found to fit the observed flavonoid content of the UAE extract with $R^{2}$ higher than 0.948, $\chi^{2}$ and RMSE lower than 0.019 and 0.085 respectively, presented in Table 5. The accuracy of the model in forecasting the experimental data can be seen in Fig. 3(iv), where the observed data and pseudo second order kinetic model fitted curves can be observed. The pseudo-second order rate model is comprehensible with a two-stage transfer of mass for the flavonoid compounds [61]. Similar findings were reported where the pseudo second order kinetic model fitted the flavonoid experimental data with higher $R^{2}>0.999$ and lower error values of average absolute relative deviation <0.38% during microwave assisted extraction of flavonoids from *Terminalia bellerica* [28]. The rate constant (g d.w./mg QE min) of the kinetic model during the extraction of flavonoids from persimmon peel was found to be 0.070, 0.095, 0.098, and 0.112 for the extract obtained at temperatures 30, 40, 50 and 60 °C respectively. The saturation concentration (mg QE/g d.w.) of flavonoids found for the same sequence of temperature was 1.560, 1.790, 2.058, and 2.350 respectively. The rise in temperature increased the rate constant and saturation concentration of the kinetic model. The higher value of the rate constant signifies the faster extraction of solute into the solvent and for the saturation concentration, it implies that the maximum amount of solute was extracted from the sample [62]. The identical trend of increase in kinetic model parameters rate constant ($s^{-1}$) and saturation concentration (g/L) from 0.317 $\times 10^{-3}$ to 0.617 $\times 10^{-3}$ and 0.826 to 1.277 was reported with an increase of temperature from 60 to 75 °C during the aqueous extraction of phenolics and flavonoids from sage leaves [63].

#### 3.2.5 Activation energy

The rate constant from the pseudo second order model was plotted against temperature and activation energy was determined using the Arrhenius equation presented in Eq. The antioxidant activity of the extract displayed a higher activation energy, as quantified by a value of 25.407 kJ/mol. Whilst, the activation energy for total phenolic content was found to be significantly lower, with a reduction of 76.521 % compared to the activation energy of antioxidant activity. The activation energy of total flavonoid content and total beta carotenoid content was found to be 12.188 kJ/mol and 12.556 kJ/mol respectively. The findings of activation energy for total phenolic content were in accordance with the results reported by Tao et al., (2014) where the activation energy for total phenolic content from red globe grapes during the UAE method of extraction was 7 kJ/mol [64]. The lower activation energy for the TPC, TFC, and TBC may be due to the thermal energy produced by the collapse of cavitation bubbles. If the Ea value is less than 20 kJ/mol, the process of extraction was regulated by diffusion; whereas if the Ea was greater than 40 kJ/mol, extraction was regulated by the reaction of solubilization. The Ea value was affected by a number of factors, such as parameters of extraction, sample, bioactive chemical and structure properties, as well as the model utilized for modeling. [65].

#### 3.2.6 Effective diffusion coefficient ($D_{e}$), mass transfer coefficient ($K_{t}$), and Biot number ($B_{i}$)

The values of $D_{e}$ and $K_{t}$ for the extracted phytochemicals from persimmon peel by ultrasonication were presented in Table 6. Temperature affects the effective diffusion coefficient and mass transfer coefficient. At a given temperature, the mass transfer coefficient was greater than the diffusion coefficient. For $Y_{B}$, the De and Kt value at an extraction temperature of 30 °C was found to be $3.187\times 10^{-11}$ and $2.087\times 10^{-06}$. With the rise of temperature from 30 °C to 40, 50, and 60 °C the Kt and De values for $Y_{B}$ was found to increase to a percentage of 18.17 and 12.01, 41.10 and 27.18, and 59.82 and 39.68 when compared with the respective coefficient values at 30 °C respectively. Similar trends of increase in $D_{e}$ and $K_{t}$ value was observed with the rise in temperature for the other responses, presented in Table 6. The values of the effective diffusion coefficient for $Y_{P}$, $Y_{A}$ and $Y_{F}$ during extraction at various temperatures was found to be in the range of $1.433\times 10^{-11}$ - $2.555\times 10^{-11}m^{2}/s$, $2.830\times 10^{-11}$ - $5.362\times 10^{-11}$ m^2^/s, and $1.766\times 10^{-11}$ - $3.912\times 10^{-11}$ $m^{2}$/s respectively.

**Table 6:** Diffusion coefficient and mass transfer coefficient for the UAE responses.

**Temperature °C**	$Y_{T}$		$Y_{A}$		$Y_{B}$		$Y_{F}$	
	$D_{e}\times 10^{-11}$	$K_{T}\times 10^{-6}$	$D_{e}\times 10^{-11}$	$K_{T}\times 10^{-6}$	$D_{e}\times 10^{-11}$	$K_{T}\times 10^{-6}$	$D_{e}\times 10^{-11}$	$K_{T}\times 10^{-6}$
30	1.433	1.330	2.830	1.935	3.187	2.087	1.766	1.472
40	1.748	1.467	3.766	2.341	3.766	2.338	2.783	1.915
50	2.111	1.621	4.760	2.771	4.497	2.655	3.158	2.077
60	2.555	1.816	5.362	3.032	5.093	2.916	3.912	2.404

The mass transfer coefficient range for $Y_{P}$ was $1.330\times 10^{-06}$ - $1.816\times 10^{-06}$, for $Y_{A}$ was $1.935\times 10^{-06}$ - $3.032\times 10^{-06}$, for $Y_{F}$ was $1.472\times 10^{-06}$ - $2.404\times 10^{-06}$. The Biot number ($B_{i}$) of $Y_{P}$, $\;Y_{A}$, $Y_{B}$ and $Y_{F}$ was found to be in the range of 10.804–14.111, 8.596–10.395, 8.702–9.956, and 9.341–12.669 respectively at a temperature range of 30 to 60 °C presented in Table 7. The Biot number of the phytochemical content of UAE permission peel extract $Y_{P}$, $\;Y_{A}$, $Y_{B}$ and $Y_{F}$ decreased with the increase in temperature.

**Table 7:** Biot number and thermodynamic parameters for the UAE responses.

Temperature, °C	Biot number	ΔH°	$\Delta S^{{}^{\circ}}$	ΔG°
30	14.111	51.975	177.321	−1.753
40	12.752			−3.526
50	11.674			−5.300
60	10.804			−7.073
30	10.395	28.083	106.943	−4.321
40	9.448			−5.390
50	8.850			−6.460
60	8.596			−7.529
30	9.956	66.896	227.280	−1.970
40	9.438			−4.242
50	8.974			−6.515
60	8.702			−8.788
30	12.669	44.486	148.646	−0.554
40	10.459			−2.040
50	9.999			−3.527
60	9.341			−5.013

### 3.3 Thermodynamic properties

Thermodynamic properties such as enthalpy, entropy, and Gibbs free energy values of UAE from persimmon fruit peel are shown in Table 7. The change in enthalpy (ΔH°) was positive indicating the process of UAE is endothermic in nature. The ΔH° values were found to be 51.975 kJ/mol, 28.083 kJ/mol, 66.896 kJ/mol, and 44.486 kJ/mol for $Y_{P}$, $\;Y_{A}$, $Y_{B}$ and $Y_{F}$ respectively. The values of the change in entropy ($\Delta S^{{}^{\circ}}$) were positive which indicates that the process of UAE is random and perturbed due to the phenomenon of cavitation. The ΔS° values of $Y_{P}$, $\;Y_{A}$, $Y_{B}$ and $Y_{F}$ were 177.321 J/mol K, 106.943 J/mol K, 227.280 J/mol K and 148.646 J/mol K respectively. The value of ΔG° for the total phenolic content of UAE persimmon peel extract was found to decrease from −1.753 to −7.073 kJ/mol with a rise of temperature from 30 to 60 °C. A similar trend of Gibbs free energy was observed with the change of temperature for the other three responses antioxidant activity, total beta carotenoid content, and total flavonoid content of UAE persimmon peel extract, presented in Table 7. Negative levels of change in Gibbs free energy (ΔG°) indicate that the extraction process is thermodynamically spontaneous. The values of ΔG° for the reactions observed to be rising with increasing temperature indicated an enhancement in extraction possibility at high temperatures.

## 4 Conclusion

The peel of the persimmon fruit was used for the extraction of phytochemicals by the application of ultrasonication. The process was modeled using ANN and optimized by integrating ANN with GA. According to the relative influence, the ultrasonication power had a positive impact on all four responses ($Y_{P}$, $Y_{A}$, $Y_{B}$ and $Y_{F}$) which signifies that with an increase in ultrasonication power, the yield of specific compounds in the extract was increasing. The solid to solvent ratio had a negative influence on antioxidant activity of UAE persimmon peel extract. The ethanol concentration and temperature of extraction had negative effect on the total beta carotenoid content of the extract. The optimum condition of the extraction process according to the integrated ANN-GA model was found to be ultrasonication power of 230.176 W, extraction temperature of 50.661 °C, solid liquid ratio of 28.273 ml/g, and solvent concentration of 62.750 % with fitness value of 3.087. The extraction data for the phytochemicals at different temperatures and optimum levels of ultrasonication power, solid to solvent ratio, and solvent concentration followed the pseudo second order kinetic model. The Gibbs free energy was found to be negative which demonstrates the thermodynamically spontaneous nature of the extraction. The outcomes of this work can be of great venture in terms of simultaneous prediction of multiple output variables, continuous data acquisition and optimizing dynamic conditions of the UAE process during industrial operation.

## CRediT authorship contribution statement

**Souvik Giri:** Writing – review & editing, Writing – original draft, Visualization, Validation, Software, Methodology, Investigation, Formal analysis, Data curation. **Kshirod Kumar Dash:** . **GVS Bhagya Raj:** Writing – review & editing, Writing – original draft, Validation, Software, Methodology, Formal analysis, Data curation. **Béla Kovács:** Writing – review & editing, Validation, Investigation. **Shaikh Ayaz Mukarram:** .

## Declaration of competing interest

The authors declare that they have no known competing financial interests or personal relationships that could have appeared to influence the work reported in this paper.
